# First-Trimester Uterine Artery Doppler Analysis in the Prediction of Later Pregnancy Complications

**DOI:** 10.1155/2015/679730

**Published:** 2015-04-20

**Authors:** Su Lynn Khong, Stefan C. Kane, Shaun P. Brennecke, Fabrício da Silva Costa

**Affiliations:** ^1^Department of Perinatal Medicine, Royal Women's Hospital, Melbourne, VIC 3052, Australia; ^2^Pauline Gandel Women's Imaging Centre, Royal Women's Hospital, Melbourne, VIC 3052, Australia; ^3^Department of Obstetrics & Gynaecology, University of Melbourne, Melbourne, VIC 3010, Australia, Australia; ^4^Monash Ultrasound for Women, Clayton, VIC 3168, Australia

## Abstract

Uterine artery Doppler waveform analysis has been extensively studied in the second trimester of pregnancy as a predictive marker for the later development of preeclampsia and fetal growth restriction. The use of Doppler interrogation of this vessel in the first trimester has gained momentum in recent years. Various measurement techniques and impedance indices have been used to evaluate the relationship between uterine artery Doppler velocimetry and adverse pregnancy outcomes. Overall, first-trimester Doppler interrogation of the uterine artery performs better in the prediction of early-onset than late-onset preeclampsia. As an isolated marker of future disease, its sensitivity in predicting preeclampsia and fetal growth restriction in low risk pregnant women is moderate, at 40–70%. Multiparametric predictive models, combining first-trimester uterine artery pulsatility index with maternal characteristics and biochemical markers, can achieve a detection rate for early-onset preeclampsia of over 90%. The ideal combination of these tests and validation of them in various patient populations will be the focus of future research.

## 1. Introduction

Most pregnancies, labours, and deliveries are normal biological processes that result in a healthy outcome for mothers and babies. Those that are not normal, however, can result in maternal and/or perinatal mortality or substantial morbidity. In the latest Centre for Maternal and Child Enquiries (CEMACE) report on maternal deaths (“Saving Mothers' Lives” 2006–2008), preeclampsia/eclampsia was the second commonest cause of direct maternal deaths in the United Kingdom (0.83 per 100,000 maternities) [1]. Preeclampsia and fetal growth restriction (FGR) have also been identified as antecedent causes in 6% and 10% of perinatal deaths, respectively. Modern antenatal care provision is focused on a risk-based approach to monitoring for adverse pregnancy outcomes such as preeclampsia, fetal growth restriction, placental abruption, and stillbirth. Increasingly, research is geared toward early identification of risks, thereby allowing early commencement of management strategies to minimise the risk of adverse outcome, including facilitation of an appropriate level of pregnancy monitoring [2]. In this review, the technique of uterine artery Doppler interrogation in the first trimester is outlined, and its role in the prediction of later pregnancy complications is discussed.

## 2. Placental Development

Implantation and trophoblastic invasion of the placenta play a crucial role in its development as an organ for the transport of nutrients and oxygen to the fetus. Placental remodelling occurs in two stages. In the first stage, between 8 and 12 weeks' gestation, trophoblastic cells invade the intradecidual portion of the spiral arteries. This is followed by deeper trophoblastic invasion into the myometrial segments of the spiral arteries from 14 weeks' gestation. The loss of smooth muscle and elastica from the spiral arteries converts the uteroplacental circulation into a low resistance, high capacitance system [3, 4]. Placental remodeling is completed by 16–18 weeks' gestation. Defective placental implantation leads to hypoperfusion, hypoxic reperfusion injury, and oxidative stress. A derangement in trophoblastic differentiation is thought to underlie the pathophysiology of gestational hypertension, preeclampsia, and fetal growth restriction (FGR). Defective implantation may also play a causative role in preterm labour, placental abruption, and second-trimester miscarriages [5, 6]. Recent studies indicate that poor placentation is associated with an imbalance of circulating vasoactive factors and, in turn, leads to maternal vascular maladaptation with associated systemic endothelial dysfunction [7–9]. Placental products are released as part of the placentation process. Levels of these biochemical markers reflect the pathophysiology of defective placentation, and, as a consequence, are assuming an increasing role in early gestation screening tests for later pregnancy complications. These biomarkers include pregnancy-associated plasma protein-A (PAPP-A), placental growth factor (PlGF), soluble fms-like tyrosine kinase-1 (sFlt-1), soluble endoglin (sEng), activin-A, and inhibin-A.

## 3. Changes in Uterine Artery Doppler Waveform in Pregnancy

In the nonpregnant state and in early pregnancy, Doppler interrogation of the uterine artery typically demonstrates low end-diastolic velocities and an early diastolic notch. Uterine artery impedance can be affected by various factors such as maternal heart rate, antihypertensive use, hormonal changes in the menstrual cycle, and chronic hyperandrogenism in the polycystic ovarian syndrome. Resistance to blood flow within the uteroplacental circulation is transmitted upstream to the uterine arteries and can be measured as an increased pulsatility index (PI) or resistance index (RI). Uterine artery PI values are affected by ethnicity and are lower in women with a high body mass index (BMI). Researchers have determined reference ranges for uterine artery Doppler parameters from 11–14 weeks' gestation to 41 weeks' gestation in various populations [10–14]. Uterine artery PI and RI values decrease with increasing gestational age, a change that is thought to be secondary to a fall in impedance in uterine vessels following trophoblastic invasion. In a prospective cross-sectional study by Gómez et al., the mean uterine artery PI continued to fall in the third trimester until week 34 [11].

“Notching” appears to be a common feature of the uterine artery Doppler waveform in pregnancy, as it is present in 46–64% of normal gestations in the first trimester. In pregnancies after 20 weeks, a diastolic notch has been defined as a fall of at least 50 cm/s from the maximum diastolic velocity [15], but most studies have utilised subjective criteria. Similar to uterine artery PI, the prevalence of notching decreases with increasing gestational age until 25 weeks' gestation and thereafter remains stable. Early diastolic notching in the uterine artery represents reduced diastolic velocities compared with those in later diastole and reflects vessel elasticity [9, 11]. Persistent early diastolic notching is thought to reflect abnormal maternal vascular tone, while defective placentation results in persistently raised uterine artery impedance [16]. Overall, notching demonstrates a low positive predictive value for preeclampsia and FGR, in contrast to its 97% negative predictive value for these conditions in a high risk study population. The poor reproducibility of uterine artery notching has led to its omission from recent research in this field, with a trend instead toward inclusion of more objective measures of vascular impedance, favouring PI. As the formula for the calculation of the PI includes the area below the waveform [(peak systolic − end-diastolic velocity)/mean velocity], the PI indirectly includes the presence or absence of an early diastolic notch.

Uterine artery Doppler analysis has the potential to predict pregnancy complications associated with uteroplacental insufficiency before the onset of clinical features. For almost 30 years, uterine artery Doppler studies have been utilized as a screening tool for uteroplacental insufficiency, mostly in the second trimester (from 18–23 + 6 weeks' gestation) [17]. Just as aneuploidy screening in the first trimester has become the accepted standard of care, so too is there an increasing impetus for the earlier prediction of other pregnancy complications, in the belief that doing so will facilitate appropriate monitoring and timely intervention to reduce maternal and/or fetal morbidity and mortality.

## 4. Measurement of Uterine Artery Doppler Parameters

Doppler assessment of uterine artery impedance can be performed between 11 + 0 and 13 + 6 weeks' gestation via a transabdominal or transvaginal approach. The transabdominal approach is the preferred method as it is less invasive with good interobserver reproducibility.

### 4.1. Transabdominal Ultrasound Technique

A 5 or 3.5-MHz curvilinear transabdominal transducer is used. A midsagittal section of the uterus and cervical canal is obtained and the transducer is moved laterally until the paracervical vessels are visualized. Color flow Doppler is applied. The uterine arteries are seen as aliasing vessels along the side of the cervix. Using pulsed wave Doppler, flow velocity waveforms from the ascending branch of the uterine artery at the point closest to the internal os are obtained, with the Doppler sampling gate set at 2 mm. Care is taken to use the smallest angle of insonation (<30°) in order to achieve the highest systolic and end-diastolic velocities. When three similar consecutive waveforms are obtained, the PI can be measured. The mean PI is calculated as the average reading from each side combined.

Another site for Doppler insonation of the uterine artery is at the level of its apparent crossover with the external iliac artery. Using this method, the probe is positioned approximately 2-3 cm inside the iliac crests and then directed toward the pelvis and the lateral side of the uterus. Color flow Doppler is used to identify each uterine artery. Pulsed wave Doppler is applied approximately 1 cm above the point at which the uterine artery crosses over the external iliac artery. This ensures that Doppler velocities are obtained from the main uterine artery trunk [18, 19]. This is similar to the technique commonly adopted for measurement of the uterine artery Doppler waveform in the second trimester.

**Figure 1:**
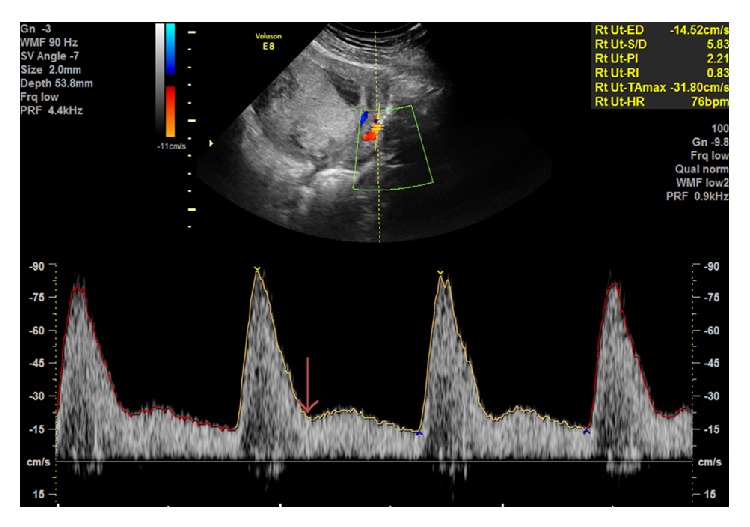
Transabdominal Doppler interrogation of the uterine artery at the level of the internal cervical os. Uterine artery waveform demonstrating raised PI with an early diastolic notch (arrow). Reproduced with permission from Associate Professor F. da Silva Costa.

Lefebvre et al. [12] compared the two different transabdominal sites of measurement in the first trimester and correlated the findings with impedance indices obtained in the second trimester at 21-22 weeks' gestation. Uterine artery PI values taken from the ascending branch at the level of internal os were higher than at the level of the apparent crossover with the external iliac artery. In addition, the former correlated better with midtrimester values. Measurements of uterine artery Doppler were easier to obtain at the level of the internal cervical os from its ascending branch, as the site of uterine artery crossover with the external iliac artery can be harder to locate with a smaller uterus in the first trimester.  Figure 1 provides an example of the transabdominal uterine artery flow velocity waveform.

### 4.2. Transvaginal Ultrasound Technique

A 4.6–8 MHz transvaginal transducer is used. The transducer is placed in the anterior vaginal fornix and a sagittal section of the cervix is obtained. The vaginal probe is then moved laterally until the paracervical vascular plexus is seen. Color flow Doppler is applied and the uterine artery is identified at the level of the cervicocorporeal junction. Measurements are taken at this point before the uterine artery branches into the arcuate arteries.

**Figure 2:**
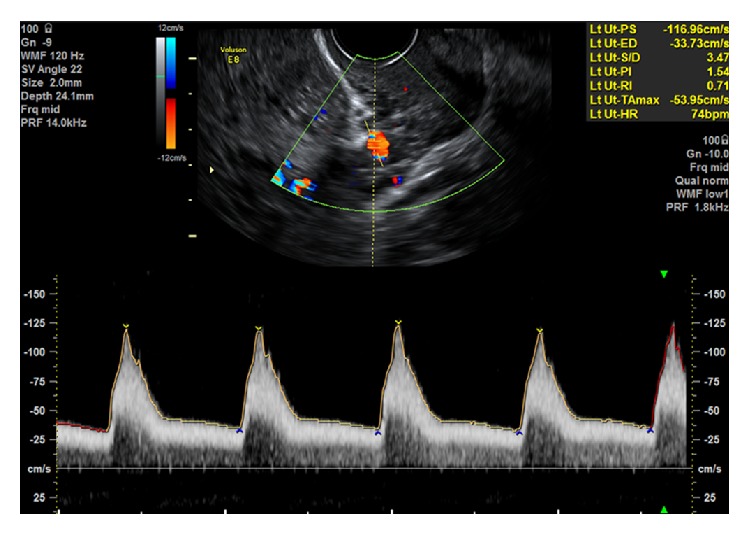
Transvaginal Doppler interrogation of the uterine artery at the cervicocorporeal junction. Normal uterine artery waveforms. Reproduced with permission from Associate Professor F. da Silva Costa.

A prospective study by Plasencia et al. found that the mean uterine artery PI at 11–13 + 6 weeks' gestation measured transabdominally was lower than that measured transvaginally: 1.83 (95% CI: 1.78–1.89) as against 1.98 (95% CI 1.93–2.08) (*p* < 0.05) [13]. Appropriate reference charts should thus be used.  Figure 2 provides an example of the transvaginal uterine artery flow velocity waveform.

## 5. Prediction of Adverse Pregnancy Outcome

Around 2–8% of pregnancies are affected by preeclampsia [20]. This condition is commonly divided into early-onset (diagnosed and requiring delivery < 34 weeks' gestation) and late-onset disease. Early-onset preeclampsia occurs less frequently (0.4–1%) than late-onset preeclampsia but is responsible for a more significant burden of disease, with its associated prematurity and fetal growth restriction (FGR), in addition to increased long-term maternal cardiovascular morbidity [8, 20–22]. Early-onset preeclampsia or “placental PE” results from impaired trophoblast invasion into the spiral arteries, causing placental ischemia and oxidative stress. Placental histology in early-onset preeclampsia or FGR often demonstrates thrombotic changes in the villous trees, lending support to this theory [20, 21, 23, 24]. On the other hand, late-onset preeclampsia or “maternal PE” is thought to be secondary to maternal cardiovascular and metabolic predisposition for endothelial dysfunction and shares similar risk factors for adult cardiac disease such as hypertension, obesity, impaired glucose tolerance, and dyslipidemia [25, 26]. The placenta in such cases may appear normal or have minimal abnormalities histopathologically. Consequently, the uterine artery Doppler parameters may remain within the normal range.

Fetal growth restriction (FGR) that develops in the absence of preeclampsia may also have its origin in defective placentation [27]. It has far reaching consequences: affected infants have an increased risk of coronary artery disease, hypertension, stroke, and diabetes in adulthood [23], in addition to increased rates of short-term morbidity and mortality. The term “small for gestational age (SGA)” has at times been used interchangeably with “fetal growth restriction” or “intrauterine growth restriction (IUGR),” even though the definition of SGA covers a spectrum ranging from constitutionally small healthy infants to those who failed to achieve their genetic growth potential and require preterm delivery.

Given the common origins of FGR and preeclampsia (especially early-onset PE) in defective placentation and consequent uteroplacental insufficiency, first-trimester Doppler assessment of the uterine circulation has been studied in numerous populations to determine its utility in the prediction of these later pregnancy complications.

### 5.1. Doppler Studies in Unselected and High Risk Pregnant Women

The predictive accuracy of second-trimester uterine artery Doppler analysis outperforms its utility in the first trimester. Studies in the first trimester vary in their reported results due to heterogeneity of vascular impedance measures, gestational age at screening, and in the prevalence and definition of preeclampsia and FGR. In addition, the performance of uterine artery Doppler velocimetry as a screening test is dependent on the pretest probability that the disease will be present in the target population.

A recent meta-analysis by Velauthar et al. [28] reviewed the accuracy of uterine artery Doppler analysis in the first trimester in the prediction of FGR and preeclampsia. Eighteen studies involving 55 974 women were evaluated, with fifteen of these studies enrolling women with low risk pregnancies. Uterine artery RI or PI ≥ 90th centile and the presence of notching (unilateral/bilateral) were used to define abnormal flow velocity waveforms (FVW). There were only two studies evaluating the role of uterine artery notching and therefore pooling estimates for prediction of preeclampsia was not feasible for this variable. An abnormal uterine artery PI in the first trimester was predictive of preeclampsia and early-onset preeclampsia with sensitivities of 26.4% and 47.8%, respectively. Fetal growth restriction was predicted at 15.4%, whereas early-onset FGR was associated with a higher sensitivity of 39.2%. The sensitivity achieved for placental abruption was 44.4%. First-trimester Doppler indices showed a low predictive accuracy for stillbirth, with a sensitivity of 14.5%.

This meta-analysis demonstrated that screening for adverse pregnancy outcome with first-trimester uterine artery Doppler analysis was comparable to screening based on maternal risk factors alone. Although the studies that evaluated early-onset disease were performed in women who were deemed low risk, the authors did not find any significant change in the estimates for secondary outcomes with notching or for any adverse composite outcome with waveform abnormalities after inclusion of studies in high risk women.

### 5.2. Sequential Testing in the First and Second Trimester of Pregnancy

Plasencia et al. examined the uterine artery PI in 3107 pregnancies at 11 + 0 to 13 + 6 weeks and compared the measurements with those at a later gestation (21 + 0 to 23 + 6 weeks). Consistent with previous research, the uterine artery PI was above the 90th centile in 77% of cases of early preeclampsia and in 27% of the late preeclampsia cases. An elevated uterine artery PI above the 90th centile persisted at 21 + 0 to 24 + 6 weeks in 94% of the early preeclampsia cases, 74% of the late preeclampsia cases, and 37% of those who did not develop preeclampsia. A predictive testing model incorporating maternal factors, uterine artery PI in the first trimester, and the change in uterine artery PI between the first and second trimesters achieved a detection rate for early preeclampsia of 90.9% at a false positive rate of 5%. The authors concluded that reserving second-trimester testing for the 20% of women with the highest risk from first-trimester screening would achieve the same detection rate. This method of contingency screening resulted in three quarters of women initially screened as high risk being reassigned to the low risk group and streamlined the remaining high risk women for increased surveillance [29].

A similar study was performed by Gómez et al. Sequential uterine artery Doppler recordings were taken at 11–14 weeks and repeated at 19–22 weeks. The mean PI was calculated from bilateral uterine artery measurements, and the presence of early diastolic notching was noted. Women whose pregnancies went on to develop complications (preeclampsia, gestational hypertension, and FGR) demonstrated a higher mean PI and persistence of a bilateral notch compared to pregnancies with normal outcomes. A persistently raised uterine artery PI > 95th centile was associated with the greatest risk of adverse outcome (OR 10.7; 95% CI 3.7–30.9). Even when the uterine artery PI normalized between the first and second trimesters, women still had a substantially increased risk of pregnancy complications (OR 5; 95% CI 2.1–10.6). Similar risks were seen in women with persistent bilateral notching [30].

### 5.3. Multiple Gestations

Although the risk of preeclampsia is increased twofold in twin gestations, the majority of studies to date have been conducted on singleton pregnancies. Svirsky et al. [31] sought to compare the distribution of mean arterial pressure (MAP) and uterine artery Doppler PI in the first trimester in 147 twin pregnancies. There was no significant difference in MAP levels between twins and singletons in women who did not go on to develop preeclampsia. Chorionicity did not affect MAP levels. The uterine Doppler PI values were statistically significantly lower in twins than in singletons, and dichorionic twins had lower PI compared with monochorionic twins. Women with twin pregnancies complicated by preeclampsia had a significantly higher MAP than women who were unaffected, but, in general, the uterine artery PI levels were significantly lower, contrary to findings from singleton studies. The authors postulated that this is due to overcompensation of blood flow to the placenta. Second-trimester studies on uterine artery Doppler in twins confirm lower PI values during the course of the pregnancy, which decrease with advancing gestational age. Uterine artery Doppler PI reference ranges for twin pregnancies should be validated in larger studies before incorporation into clinical practice [32].

### 5.4. Multifactorial Approach with Biophysical and Biochemical Markers

#### 5.4.1. Preeclampsia

Women at risk of adverse pregnancy outcomes are generally identified based on their clinical history [18, 33]. Screening by maternal history alone will detect a third of women who will develop preeclampsia but is ineffective in nulliparous women, who are at particular risk of this complication. In a prospective study involving 8366 singleton pregnancies, Poon et al. investigated the role of uterine artery Doppler in the development of a predictive model for prediction of early preeclampsia. The detection rates for early preeclampsia, late preeclampsia, and gestational hypertension achieved by a model incorporating clinical history (history of preeclampsia, chronic hypertension, and method of conception) and maternal demographics (age, BMI, and ethnicity) alone were 47%, 41%, and 31%, respectively, at a 10% false positive rate. In women who subsequently developed preeclampsia or gestational hypertension, the lowest, mean, and highest uterine artery PI were significantly higher. First-trimester uterine artery Doppler improved the detection rate of early preeclampsia to 81%, with smaller improvements to 45% and 35% for late preeclampsia and gestational hypertension, respectively. Patient specific risk for early and late preeclampsia and gestational hypertension could be calculated based on maternal-factor-derived* a priori* risk and the lowest uterine artery PI value using a multivariate regression model [34], although other authors have found no significant difference in lower, mean, and higher uterine artery resistance indices in screening sensitivity for the prediction of preeclampsia [35]. Including mean arterial pressure led to a further increase in detection rates of early and late preeclampsia and gestational hypertension to 89%, 57%, and 50%, respectively [36].

Multiple biochemical markers have been studied individually and in combination as potential markers for adverse pregnancy outcomes. As first-trimester combined screening for fetal aneuploidy has been widely adopted, the biochemical markers used in this testing—PAPP-A and free *β*hCG—have been evaluated extensively. Other disease markers proposed for the prediction of preeclampsia and other adverse pregnancy outcomes include soluble endoglin (sEng), soluble fms-like tyrosine kinase-1 (sFlt-1), placental growth factor (PlGF), inhibin-A, activin-A, a disintegrin and metalloprotease 12 (ADAM12), and placental protein 13 (PP13), as outlined in Table 1 [37].

Despite the clear association between individual biomarkers (including uterine artery Doppler parameters) and preeclampsia risk, none has demonstrated sufficient sensitivity and specificity to perform as a screening test in isolation. Adequate screening test performance has only been achieved through the use of multiparametric testing regimens. The landmark study that identified the value of this approach was published by Poon et al. in 2009 [38], in which an algorithm incorporating maternal history, uterine artery PI (UtA-PI), mean arterial pressure (MAP), PAPP-A, and PlGF achieved a detection rate for early preeclampsia of 93% at a 5% false positive rate. Since then, numerous other algorithms have been developed in various patient populations worldwide, as outlined in Table 2.

The external validity of some of these multiparametric models for the prediction of preeclampsia has been evaluated in several recent studies, with algorithms applied to populations differing in ethnicity and background incidence of the disease. The performance of these models for the prediction of late and early preeclampsia is summarised in Tables 3 and 4, respectively.

The lack of reproducibility demonstrated in the studies above highlights differences in the prevalence of preeclampsia and risk profile present in various patient populations, which in turn directly influences the positive and negative predictive values of predictive algorithms. For example, the study by Farina et al. [39] was conducted in a population with a baseline incidence of preeclampsia of 7%, which is higher than that reported by most centres in the developed world [40]. In addition, predictive models developed using logistic regression methods in one population may not be directly applicable to other populations. The limitations of external validation of the various predictive models further reinforce the theory that early and late preeclampsia are two distinct disease entities and demonstrate that a single model is unlikely to be an effective predictive tool in all settings.

The novel technology of cell-free fetal DNA testing in maternal serum is commonly used for prenatal aneuploidy screening but may demonstrate other predictive applications. Higher levels of cffDNA have been found in women who develop preeclampsia and are thought to be secondary to accelerated placental apoptosis from hypoxia and oxidative stress in placental insufficiency [41]. Significantly elevated levels are seen in early-onset or severe preeclampsia and precede any clinical symptoms. Maternal ethnicity, BMI, and smoking status are known to affect cffDNA levels, but these confounding factors have not been adequately controlled for in earlier studies. Rolnik et al. demonstrated an increase in median total cell-free DNA and a decrease in median fetal fraction at 11–13 weeks in women who subsequently developed early-onset preeclampsia [42]. However, the results were not statistically significant once converted to multiples of median and adjusted for maternal characteristics, and the authors concluded that cell-free fetal DNA levels are not predictive of preeclampsia in isolation.

#### 5.4.2. Fetal Growth Restriction

As noted earlier, preeclampsia and fetal growth restriction share elements of a common origin in deficient placentation. It is thus not surprising that many of the algorithmic approaches to the prediction of the former have also been studied in early pregnancy screening for the latter. Researchers from the Fetal Medicine Foundation (UK) devised a predictive model for SGA (defined as birthweight less than the 5th%) that incorporated maternal factors and numerous biomarkers, including mean arterial pressure (MAP), fetal nuchal translucency (NT) thickness, free beta-human chorionic gonadotrophin (beta-hCG), serum pregnancy-associated plasma protein-A (PAPP-A), uterine artery pulsatility index (PI), placental growth factor (PlGF), placental protein 13 (PP13), and a disintegrin and metalloprotease 12 (ADAM12). This model achieved a 73% detection rate for SGA requiring delivery prior to 37 weeks' gestation, at a false positive rate of 10% [43]. A subsequent study from this centre used maternal characteristics, uterine artery pulsatility index, mean arterial pressure, serum pregnancy-associated plasma protein-A, and placental growth factor to predict 52.3% of preterm SGA at a false positive rate (FPR) of 10% [44].

More recently, Crovetto et al. [45] used a two-tier model to screen for early SGA: serum PAPP-A and free *β*hCG at 8–10 weeks in combination with uterine artery PI at 11–13 + 6 weeks. Maternal* a priori* risk factors, including MAP, were included in the assessment. The prevalence of early and late SGA was 0.6% and 7.9%, respectively, in the cohort of 4970 women. Sixty-seven percent of women with early SGA had superimposed preeclampsia compared to 8% in the late SGA group. The detection rate for early SGA was 75% (FPR 10%), although the detection rate was only 30% in the absence of preeclampsia. The detection rate for late SGA was 31.3% and 22.3% for cases with and without preeclampsia. The investigators concluded that the performance of first-trimester screening for early SGA was strongly influenced by concomitant preeclampsia. Even though the detection rate for late SGA was low, first-trimester screening may help identify a group of women who would benefit from fetal growth ultrasonography in the third trimester [45].

## 6. Clinical Implications of Screening

The early prediction of later pregnancy adverse outcomes permits the initiation of management strategies that may prevent or mitigate these complications. The role of aspirin in preventing preeclampsia and adverse pregnancy outcomes has been the subject of a large number of trials. The latest Cochrane review, published in 2007 [46], founda 17% reduction in the risk of preeclampsia associated with the use of antiplatelet agents (46 trials involving 32,891 women, relative risk [RR] 0.83, and 95% CI 0.77–0.89), with a number needed to treat (NNT) of 72 (95% CI 52–119). Antiplatelet agents resulted in a greater risk reduction of preeclampsia for high risk women (risk difference [RD] −5.2% [95% CI −7.5 to −2.9], NNT 19) compared with moderate risk women (RD −0.84 [95% CI −1.37 to −0.3], NNT 119). In addition, antiplatelet use was associated with a 10% reduction in SGA (36 trials, 23,638 women, RR 0.90, and 95% CI 0.83–0.98) as well as an 8% reduction in the relative risk of preterm birth (29 trials, 31,151 women, RR 0.92, and 95% CI 0.88–0.97, NNT 72). Lastly, there was a 14% reduction in fetal or neonatal deaths (40 trials, 33,098 women, RR 0.86, 95% CI 0.76–0.98, and NNT 243).

In a meta-analysis by Bujold et al. reviewing 27 randomised controlled trials involving 11 348 women, low dose aspirin started at 16 weeks' gestation or earlier resulted in a greater reduction in preeclampsia and fetal growth restriction (RR 0.47 and 0.44, resp.) than later commencement [47]. The risk of severe preeclampsia was also significantly reduced (RR 0.09), and, compared to controls, there was a 78% reduction in preterm birth. Velauthar et al. [28] estimated the NNT for aspirin to prevent early-onset preeclampsia to be 1000–2500, based on a baseline population prevalence of 0.4–1%. In women with abnormal first-trimester uterine artery Doppler waveforms, however, the NNT for aspirin is lowered significantly (to 143–421).

At present, most interventions in pregnancy are based on intensive maternal and fetal monitoring. The traditional model of antenatal care often has women undertake their initial appointment at 16 weeks, with subsequent antenatal visits spaced more closely with advancing gestation. Current advances in screening have led to a proposal for this model to be modified to permit risk stratification of women from as early as 11–13 weeks. Earlier screening in pregnancy would allow women at a higher risk to be monitored accordingly, participate in early intervention trials, and commence prophylactic therapy. It would also permit the judicious allocation of limited resources [48–51].

## 7. Conclusions

The predictive accuracy of first-trimester uterine artery Doppler is better in the detection of early-onset preeclampsia and FGR than late-onset disease. The sensitivities and specificities of uterine artery Doppler indices for the prediction of preeclampsia in low risk populations vary from 34% to 76% and 83% to 93%, respectively. The low sensitivity of this test limits its utility as a disease marker in isolation. There is growing evidence that multiparametric models in the first trimester have the potential to improve detection rates for preeclampsia and other adverse pregnancy outcomes. Algorithms that combine maternal characteristics, uterine artery Doppler velocimetry, and biochemical markers in the first trimester have the potential to improve the detection rate of early-onset preeclampsia to over 90% at a false positive rate of 10%. Further research is required to evaluate the generalizability of multiparametric models in different resource settings, in addition to assessing the impact of screening on clinical outcomes.

## Figures and Tables

**Table 1:** First-trimester biochemical biomarkers associated with preeclampsia (PE).

Marker	Mechanism of action	Levels associated with PE
PAPP-A	Insulin-like growth factor binding protein protease: impaired trophoblast invasion and fetal cell growth	↓

PlGF	Vascular endothelial growth factor trophoblastic proliferation and implantation	↓

sFlt-1	Antiangiogenic factor	↑

Inhibin-A & activin-A	Maintenance of spiral artery function	↓

sEng	Impairs binding of transforming growth-*β*1 to cell surface receptors, inhibiting angiogenesis	↑

PP13	Binds to protein on extracellular matrix between placenta and myometrium: placenta implantation & remodeling	↓

ADAM12	Placenta derived multidomain glycoprotein: fetal & placental growth	↓

**Table 2:** Detection rate (DR) of early preeclampsia at a 10% false positive rate using various multiparametric predictive models (i.e., those including maternal characteristics, uterine artery Doppler, and biochemical markers).

Predictive model	Parameters	DR%
Parra-Cordero [52]	BMI, smoking, lowest UtA-PI, and PlGF	47

Odibo [53]	HTN, mean UtA-PI, PAPP-A, and PP-13	68

Poon [54]	Maternal history, UtA-PI, and PAPP-A	71.9

Scazzocchio [55]	Ethnicity, BMI, parity, previous PE, age, HTN, renal disease, MAP, and mean UtA-PI	81

Poon [36]	Ethnicity, BMI, parity, previous PE, age, HTN, DM, thrombophilia, smoking, MAP, and lowest UtA-PI	89

Crovetto [56]	Maternal characteristics, MAP, UtA-PI, and sFlt-1	91.2

Poon [57]	Maternal characteristics, lowest UtA-PI, MAP, and PlGF	92.3

Poon [38]	Maternal factors, UtA-PI, MAP, PAPP-A, and PlGF	93.1^*^

Poon [58]	Ethnicity, BMI, parity, previous PE, age, HTN, DM, thrombophilia, smoking, MAP, lowest UtA-PI, and PAPP-A	95

Akolekar [59]	Maternal factors, MAP, UtA-PI, PAPP-A, PlGF, PP13, sEng, inhibin-A, activin-A, PTX3, and P-selectin	95.2

Akolekar [60]	UtA-PI, MAP, PAPP-A, and PlGF	96.3

^*^This study reported detection rates at a 5% false positive rate.

**Table 3:** External validation of multiparametric models for the prediction of late preeclampsia (>34 weeks).

Study	Population	Incidence of late preeclampsia	Predictive models tested	Detection rate (%) at 10% false positive rate	
				Reported	Observed
			Poon [38] (lowest UtA-PI)	45.3	38.5
			Poon [38] (mean UtA-PI)	46.9	41
Farina et al. [39]	554 Mediterranean women	7%	Poon [38] (highest UtA-PI)	46.1	43.6
			Poon [54]	41.1	35.9
			Poon [58]	57	84.6

Oliveira et al. [61]	2962 American women	4.1–5%	Parra-Cordero [52] Scazzocchio [55]	29 40	18 31

Park et al. [62]	3066 Australian women	2.3%	Poon [58]	57	35.2

Skråstad et al. [63]	541 nulliparous Norwegian women	3.7%	Akolekar [60] PREDICTOR posterior^*^	N/A N/A	30 30

UtA-PI = uterine artery pulsatility index.

^*^Proprietary predictive model (PerkinElmer, Waltham, MA, USA) incorporating BMI, ethnicity, parity, family history of preeclampsia, chronic hypertension, MAP, UtA lowest PI, PlGF, and PAPP-A.

**Table 4:** External validation of multiparametric models for the prediction of early preeclampsia (<34 weeks).

Study	Population	Incidence of early preeclampsia	Predictive models tested	Detection rate (%) at 10% false positive rate	
				Reported	Observed
Oliveira et al. [61]	2962 American women	1–1.2%	Parra-Cordero [52] Scazzocchio [55] Poon [58] Odibo [53]	47 81 95 68	29 43 52 80

Park et al. [62]	3066 Australian women	0.4%	Poon [58]	95	91.7

## References

[1] Cantwell R., Clutton-Brock T., Cooper G. (2011). Saving Mothers' Lives: reviewing maternal deaths to make motherhood safer: 2006–2008. The Eighth Report of the Confidential Enquiries into Maternal Deaths in theThe eighth report of the confidential enquiries into maternal deaths in the United Kingdom. *BJOG*.

[2] Kane S. C., Da Silva Costa F., Brennecke S. (2014). First trimester biomarkers in the prediction of later pregnancy complications. *BioMed Research International*.

[3] Wallace A. E., Host A. J., Whitley G. S., Cartwright J. E. (2013). Decidual natural killer cell interactions with trophoblasts are impaired in pregnancies at increased risk of preeclampsia. *The American Journal of Pathology*.

[4] Wallace A. E., Whitley G. S., Thilaganathan B., Cartwright J. E. (2015). Decidual natural killer cell receptor expression is altered in pregnancies with impaired vascular remodeling and a higher risk of pre-eclampsia. *Journal of Leukocyte Biology*.

[5] Brosens I., Pijnenborg R., Vercruysse L., Romero R. (2011). The ‘great Obstetrical Syndromes’ are associated with disorders of deep placentation. *American Journal of Obstetrics and Gynecology*.

[6] Carbillon L., Challier J. C., Alouini S., Uzan M., Uzan S. (2001). Uteroplacental circulation development: doppler assessment and clinical importance. *Placenta*.

[7] Everett T. R., Lees C. C. (2012). Beyond the placental bed: placental and systemic determinants of the uterine artery Doppler waveform. *Placenta*.

[8] Tuuli M. G., Odibo A. O. (2010). First- and second-trimester screening for preeclampsia and intrauterine growth restriction. *Clinics in Laboratory Medicine*.

[9] Zhong Y., Tuuli M., Odibo A. O. (2010). First-trimester assessment of placenta function and the prediction of preeclampsia and intrauterine growth restriction. *Prenatal Diagnosis*.

[10] Alves J. A. G., Silva B. Y. D. C., Sousa P. C. P. D., Maia S. B., Costa F. D. S. (2013). Reference range of uterine artery Doppler parameters between the 11th and 14th pregnancy weeks in a population sample from Northeast Brazil. *Revista Brasileira de Ginecologia e Obstetrícia*.

[11] Gómez O., Figueras F., Fernández S. (2008). Reference ranges for uterine artery mean pulsatility index at 11–41 weeks of gestation. *Ultrasound in Obstetrics & Gynecology*.

[12] Lefebvre J., Demers S., Bujold E. (2012). Comparison of two different sites of measurement for transabdominal uterine artery Doppler velocimetry at 11–13 weeks. *Ultrasound in Obstetrics and Gynecology*.

[13] Plasencia W., Barber M. A., Alvarez E. E., Segura J., Valle L., Garcia-Hernandez J. A. (2011). Comparative study of transabdominal and transvaginal uterine artery doppler pulsatility indices at 11–13 + 6 weeks. *Hypertension in Pregnancy*.

[14] Ridding G., Schluter P. J., Hyett J. A., McLennan A. C. (2014). Uterine artery pulsatility index assessment at 11–13 weeks' gestation. *Fetal Diagnosis & Therapy*.

[15] Sciscione A. C., Hayes E. J. (2009). Uterine artery doppler flow studies in obstetric practice. *American Journal of Obstetrics & Gynecology*.

[16] Mo L. Y. L., Bascom P. A. J., Ritchie K., McCowan L. M. E. (1988). A transmission line modelling approach to the interpretation of uterine Doppler waveforms. *Ultrasound in Medicine & Biology*.

[17] Cnossen J. S., Morris R. K., Ter Riet G. (2008). Use of uterine artery Doppler ultrasonography to predict pre-eclampsia and intrauterine growth restriction: a systematic review and bivariable meta-analysis. *Canadian Medical Association Journal*.

[18] National Institute for Health and Clinical Excellence (2010). *Guidance: Hypertension in Pregnancy: The Management of Hypertensive Disorders During Pregnancy*.

[19] Khalil A., Nicolaides K. H. (2013). How to record uterine artery Doppler in the first trimester. *Ultrasound in Obstetrics & Gynecology*.

[20] Steegers E. A. P., von Dadelszen P., Duvekot J. J., Pijnenborg R. (2010). Pre-eclampsia. *The Lancet*.

[21] Huppertz B. (2008). Placental origins of preeclampsia: challenging the current hypothesis. *Hypertension*.

[22] Visser G. H. A., Bilardo C. M., Lees C. (2014). Fetal growth restriction at the limits of viability. *Fetal Diagnosis & Therapy*.

[23] de Boo H. A., Harding J. E. (2006). The developmental origins of adult disease (Barker) hypothesis. *Australian & New Zealand Journal of Obstetrics and Gynaecology*.

[24] Gómez O., Martínez J. M., Figueras F. (2005). Uterine artery Doppler at 11-14 weeks of gestation to screen for hypertensive disorders and associated complications in an unselected population. *Ultrasound in Obstetrics & Gynecology*.

[25] Melchiorre K., Sharma R., Thilaganathan B. (2014). Cardiovascular implications in preeclampsia: an overview. *Circulation*.

[26] Verlohren S., Melchiorre K., Khalil A., Thilaganathan B. (2014). Uterine artery Doppler, birth weight and timing of onset of pre-eclampsia: providing insights into the dual etiology of late-onset pre-eclampsia. *Ultrasound in Obstetrics & Gynecology*.

[27] Figueras F., Gratacós E. (2014). Update on the diagnosis and classification of fetal growth restriction and proposal of a stage-based management protocol. *Fetal Diagnosis & Therapy*.

[28] Velauthar L., Plana M. N., Kalidindi M. (2014). First-trimester uterine artery Doppler and adverse pregnancy outcome: a meta-analysis involving 55 974 women. *Ultrasound in Obstetrics & Gynecology*.

[29] Plasencia W., Maiz N., Poon L., Yu C., Nicolaides K. H. (2008). Uterine artery Doppler at 11 + 0 to 13 + 6 weeks and 21 + 0 to 24 + 6 weeks in the prediction of pre-eclampsia. *Ultrasound in Obstetrics & Gynecology*.

[30] Gómez O., Figueras F., Martínez J. M. (2006). Sequential changes in uterine artery blood flow pattern between the first and second trimesters of gestation in relation to pregnancy outcome. *Ultrasound in Obstetrics and Gynecology*.

[31] Svirsky R., Yagel S., Ben-Ami I., Cuckle H., Klug E., Maymon R. (2014). First trimester markers of preeclampsia in twins: maternal mean arterial pressure and uterine artery Doppler pulsatility index. *Prenatal Diagnosis*.

[32] Geipel A., Hennemann F., Fimmers R. (2011). Reference ranges for Doppler assessment of uterine artery resistance and pulsatility indices in dichorionic twin pregnancies. *Ultrasound in Obstetrics and Gynecology*.

[33] Duckitt K., Harrington D. (2005). Risk factors for pre-eclampsia at antenatal booking: systematic review of controlled studies. *British Medical Journal*.

[34] Poon L. C. Y., Staboulidou I., Maiz N., Plasencia W., Nicolaides K. H. (2009). Hypertensive disorders in pregnancy: screening by uterine artery Doppler at 11–13 weeks. *Ultrasound in Obstetrics & Gynecology*.

[35] Napolitano R., Rajakulasingam R., Memmo A., Bhide A., Thilaganathan B. (2011). Uterine artery Doppler screening for pre-eclampsia: comparison of the lower, mean and higher first-trimester pulsatility indices. *Ultrasound in Obstetrics & Gynecology*.

[36] Poon L. C. Y., Karagiannis G., Leal A., Romero X. C., Nicolaides K. H. (2009). Hypertensive disorders in pregnancy: screening by uterine artery Doppler imaging and blood pressure at 11–13 weeks. *Ultrasound in Obstetrics & Gynecology*.

[37] Allen R. E., Rogozinska E., Cleverly K., Aquilina J., Thangaratinam S. (2014). Abnormal blood biomarkers in early pregnancy are associated with preeclampsia: a meta-analysis. *European Journal of Obstetrics & Gynecology and Reproductive Biology*.

[38] Poon L. C. Y., Kametas N. A., Maiz N., Akolekar R., Nicolaides K. H. (2009). First-trimester prediction of hypertensive disorders in pregnancy. *Hypertension*.

[39] Farina A., Rapacchia G., Freni Sterrantino A., Pula G., Morano D., Rizzo N. (2011). Prospective evaluation of ultrasound and biochemical-based multivariable models for the prediction of late pre-eclampsia. *Prenatal Diagnosis*.

[40] Thornton C., Dahlen H., Korda A., Hennessy A. (2013). The incidence of preeclampsia and eclampsia and associated maternal mortality in Australia from population-linked datasets: 2000–2008. *American Journal of Obstetrics & Gynecology*.

[41] Martin A., Krishna I., Martina B., Samuel A. (2014). Can the quantity of cell-free fetal DNA predict preeclampsia: a systematic review. *Prenatal Diagnosis*.

[42] Rolnik D. L., O'Gorman N., Fiolna M., van den Boom D., Nicolaides K. H., Poon L. C. (2015). Maternal plasma cell-free DNA in the prediction of pre-eclampsia. *Ultrasound in Obstetrics & Gynecology*.

[43] Karagiannis G., Akolekar R., Sarquis R., Wright D., Nicolaides K. H. (2011). Prediction of small-for-gestation neonates from biophysical and biochemical markers at 11–13 weeks. *Fetal Diagnosis and Therapy*.

[44] Poon L. C. Y., Syngelaki A., Akolekar R., Lai J., Nicolaides K. H. (2013). Combined screening for preeclampsia and small for gestational age at 11–13 weeks. *Fetal Diagnosis and Therapy*.

[45] Crovetto F., Crispi F., Scazzocchio E. (2014). First-trimester screening for early and late small-for-gestational-age neonates using maternal serum biochemistry, blood pressure and uterine artery Doppler. *Ultrasound in Obstetrics and Gynecology*.

[46] Duley L., Henderson-Smart D. J., Meher S., King J. F. (2007). Antiplatelet agents for preventing pre-eclampsia and its complications (review). *The Cochrane Database of Systematic Reviews*.

[47] Bujold E., Roberge S., Lacasse Y. (2010). Prevention of preeclampsia and intrauterine growth restriction with aspirin started in early pregnancy a meta-analysis. *Obstetrics & Gynecology*.

[48] Cnossen J. S., Riet G. T., Mol B. W. (2009). Are tests for predicting pre-eclampsia good enough to make screening viable? A review of reviews and critical appraisal. *Acta Obstetricia et Gynecologica Scandinavica*.

[49] Costa S. L., Proctor L., Dodd J. M. (2008). Screening for placental insufficiency in high-risk pregnancies: is earlier better?. *Placenta*.

[50] Nicolaides K. H. (2011). Turning the pyramid of prenatal care. *Fetal Diagnosis and Therapy*.

[51] Poon L. C., Nicolaides K. H. (2014). Early prediction of preeclampsia. *Obstetrics and Gynecology International*.

[52] Parra-Cordero M., Rodrigo R., Barja P. (2013). Prediction of early and late pre-eclampsia from maternal characteristics, uterine artery Doppler and markers of vasculogenesis during first trimester of pregnancy. *Ultrasound in Obstetrics & Gynecology*.

[53] Odibo A. O., Zhong Y., Goetzinger K. R. (2011). First-trimester placental protein 13, PAPP-A, uterine artery Doppler and maternal characteristics in the prediction of pre-eclampsia. *Placenta*.

[54] Poon L. C. Y., Maiz N., Valencia C., Plasencia W., Nicolaides K. H. (2009). First-trimester maternal serum pregnancy-associated plasma protein-A and pre-eclampsia. *Ultrasound in Obstetrics & Gynecology*.

[55] Scazzocchio E., Figueras F., Crispi F. (2013). Performance of a first-trimester screening of preeclampsia in a routine care low-risk setting. *American Journal of Obstetrics and Gynecology*.

[56] Crovetto F., Figueras F., Triunfo S. (2015). First trimester screening for early and late preeclampsia based on maternal characteristics, biophysical parameters and angiogenic factors. *Prenatal Diagnosis*.

[57] Poon L. C. Y., Akolekar R., Lachmann R., Beta J., Nicolaides K. H. (2010). Hypertensive disorders in pregnancy: screening by biophysical and biochemical markers at 11–13 weeks. *Ultrasound in Obstetrics and Gynecology*.

[58] Poon L. C. Y., Stratieva V., Piras S., Piri S., Nicolaides K. H. (2010). Hypertensive disorders in pregnancy: combined screening by uterine artery Doppler, blood pressure and serum PAPP-A at 11–13 weeks. *Prenatal Diagnosis*.

[59] Akolekar R., Syngelaki A., Sarquis R., Zvanca M., Nicolaides K. H. (2011). Prediction of early, intermediate and late pre-eclampsia from maternal factors, biophysical and biochemical markers at 11–13 weeks. *Prenatal Diagnosis*.

[60] Akolekar R., Syngelaki A., Poon L., Wright D., Nicolaides K. H. (2013). Competing risks model in early screening for preeclampsia by biophysical and biochemical markers. *Fetal Diagnosis and Therapy*.

[61] Oliveira N., Magder L. S., Blitzer M. G., Baschat A. A. (2014). First-trimester prediction of pre-eclampsia: external validity of algorithms in a prospectively enrolled cohort. *Ultrasound in Obstetrics & Gynecology*.

[62] Park F. J., Leung C. H. Y., Poon L. C. Y., Williams P. F., Rothwell S. J., Hyett J. A. (2013). Clinical evaluation of a first trimester algorithm predicting the risk of hypertensive disease of pregnancy. *Australian and New Zealand Journal of Obstetrics and Gynaecology*.

[63] Skråstad R., Hov G., Blaas H., Romundstad P., Salvesen K. (2014). Risk assessment for preeclampsia in nulliparous women at 11–13 weeks gestational age: prospective evaluation of two algorithms. *British Journal of Obstetrics and Gynaecology*.

